# Short-Term Combined Exercise Improves Inflammatory Profile in the Retina of Obese Mice

**DOI:** 10.3390/ijms21176099

**Published:** 2020-08-24

**Authors:** Thaís Dantis Pereira de Campos, Kellen Cristina da Cruz Rodrigues, Rodrigo Martins Pereira, Ana Paula Morelli, Alisson Luiz da Rocha, Raphael dos Santos Canciglieri, Adelino Sanchez Ramos da Silva, Eduardo Rochete Ropelle, José Rodrigo Pauli, Fernando Moreira Simabuco, Dennys Esper Cintra, Leandro Pereira de Moura

**Affiliations:** 1 Exercise Celular Biology Lab, School of Applied Sciences, University of Campinas, 13484–350 Limeira, Brazil; thais.dantis@gmail.com (T.D.P.d.C.); kellen.rodrigues.nut@gmail.com (K.C.d.C.R.); rodrigo_mpereira@hotmail.com (R.M.P.); rcanciglieri@gmail.com (R.d.S.C.); eduardo.ropelle@fca.unicamp.br (E.R.R.); jose.pauli@fca.unicamp.br (J.R.P.); 2 Laboratory of Molecular Biology of Exercise, School of Applied Sciences, University of Campinas, 13484–350 Limeira, Brazil; 3 Multidisciplinary Laboratory of Food and Health, Faculty of Applied Sciences, University of Campinas, 13484–350 Limeira, Brazil; apm.morelli@gmail.com (A.P.M.); simabuco@gmail.com (F.M.S.); 4 School of Physical Education and Sport of Ribeirão Preto, University of São Paulo, 14030–680 Ribeirão Preto, Brazil; alisson.rocha@usp.br (A.L.d.R.); adelinosanchez@usp.br (A.S.R.d.S.); 5 Laboratory of Nutritional Genomics, School of Applied Sciences, University of Campinas, 13484–350 Limeira, Brazil; dennys.cintra@fca.unicamp.br

**Keywords:** obesity, inflammation, insulin resistance, exercise, combined exercise, retina

## Abstract

Excess of adipose tissue increases the concentration of proinflammatory cytokines, triggering a subclinical inflammatory condition. This inflammatory profile contributes to retina damage, which can lead to retinal dysfunction and reduced vision. Regularly practicing both aerobic and strength exercises is well known for promoting anti-inflammatory effects on different organs in the peripheral and central regions. However, the effects of combined physical exercise (CPE; strength + aerobic) on the inflammatory process in the retina tissue are not yet known. This study aimed to investigate the effects of CPE on the inflammatory profile of the retina in obese mice. Swiss mice were distributed into control, sedentary obese, and trained obese groups. The trained obese group was subjected to short-term CPE, 1 h/day, for 7 days. The CPE was composed of aerobic and strength exercises in the same exercise session. The strength exercise protocol consisted of 10 climbing series, with 12 ± 1 dynamic climbing movements at 70% of the maximum voluntary carrying capacity (MVCC), and the aerobic exercise protocol consisted of 30 min of treadmill running, with an intensity of 75% of the exhaust velocity. Subsequently, the retina was excised and analyzed by Western blot. Obese animals presented impairment on glucose homeostasis and elevated levels of proinflammatory proteins in the serum and retina; however, CPE was effective in reversing these parameters, independently of changes in body adiposity. Therefore, for the first time, we have shown that short-term CPE can be an important strategy to treat an inflammatory profile in the retina.

## 1. Introduction

Currently, 1.3 billion people worldwide are overweight or obese [1]. The main consequence of obesity is the development of low-grade systemic inflammation that may contribute to the onset of diabetic retinopathy [2]. This consequence is one of the predominant causes of visual impairment, which affects around 4.2 million people worldwide [3].

The excess of adipose tissue found in obesity is well known for secreting proinflammatory cytokines. This inflammatory profile is capable of affecting different signaling pathways in both the peripheral and central systems, causing various metabolic losses in the obese individual. In the retina, when the inflammatory cytokines interleukin-1 beta (IL-1β), tumor necrosis factor-alpha (TNF-α), and the vascular endothelial growth factor (VEGF) produced by the toll-like receptor/nuclear factor kappa B (TLR4/NFκB) pathway bind to their receptors, there is an increase in the transcription of proinflammatory and pro-apoptotic genes, augmenting vascular permeability, apoptosis, and neurodegeneration [4]. In this scenario, IL-1β specifically acts as a potent mediator in the pathogenesis of ocular diseases, since it promotes inflammation, apoptosis, and extracellular matrix accumulation [4,5], contributing to the emergence of various diseases. Furthermore, VEGF, the end product of the IL-1β pathway, induces angiogenesis, and this condition increases intraocular inflammation through vessel permeability [6].

On the other hand, physical exercise acts as a Swiss army knife in controlling the systemic metabolic disorders induced by obesity in peripheral tissues such as muscle [7] and liver [8], as well as the central nervous system, such as the hypothalamus [9]. Moreover, physical exercise has anti-inflammatory properties by increasing interleukin-6 (IL-6), which, in turn, elevates interleukin-10 (IL-10) [7]. In addition, IL-10 attenuates inflammation by inhibiting IκB kinase (IKK), the interleukin-1 receptor antagonist (IL-1ra), and the soluble TNF receptor (TNFsr). In this sense, IL-10 can decrease the synthesis of proinflammatory cytokines such as TNF-α, IL-6, and IL-1β [7].

The American College of Sports Medicine recommends practicing both aerobic and resistance exercises since they activate different pathways and promote several health improvements [10]. Theodorou and colleagues showed that 8 months of combined physical exercise caused significant benefits in terms of muscle strength gain and improvements in lipid, apolipoprotein, and inflammation profile [11]. Dieli and colleagues observed that combined exercise improved body composition and reduced cardiometabolic biomarkers and systemic inflammation, with decreased secretion of proinflammatory cytokines after 16 weeks of the intervention [12].

In this context, few studies have evaluated the effect of exercise on the retina. Findings in the literature demonstrate that physical exercise on a treadmill can reduce oxidative stress [13]. Aerobic exercise is also effective in reducing deficits in retinal function and photoreceptor cell death [14] and protecting against intraocular pressure injury [15]. However, there is no study evaluating the effects of combined exercise on the retina or on the inflammatory process in this tissue.

Moreover, few studies have aimed to evaluate biomolecular aspects in specific tissues after combined exercise. Most studies have reported the benefits of exercise in terms of metabolic parameters; however, these effects are mainly linked to body weight reduction promoted by physical exercise. In this sense, to evaluate the direct effects of physical exercise on the retina, regardless of changes in body fatness caused by exercise, this study aims to evaluate whether seven sessions of combined exercise (resistance exercise (70% of maximal loading load) + aerobic exercise (75% of maximum power)) can exert any response at the ocular level. More specifically, it seeks to verify if this protocol can modulate proinflammatory cytokine levels in the retina, without the interference of changes in body composition. Therefore, this study aimed to investigate the effect of short-term combined exercise on the inflammatory profile in the retina of obese mice.

## 2. Results

### 2.1. Physiological and Metabolic Parameters

**Figure 1:**
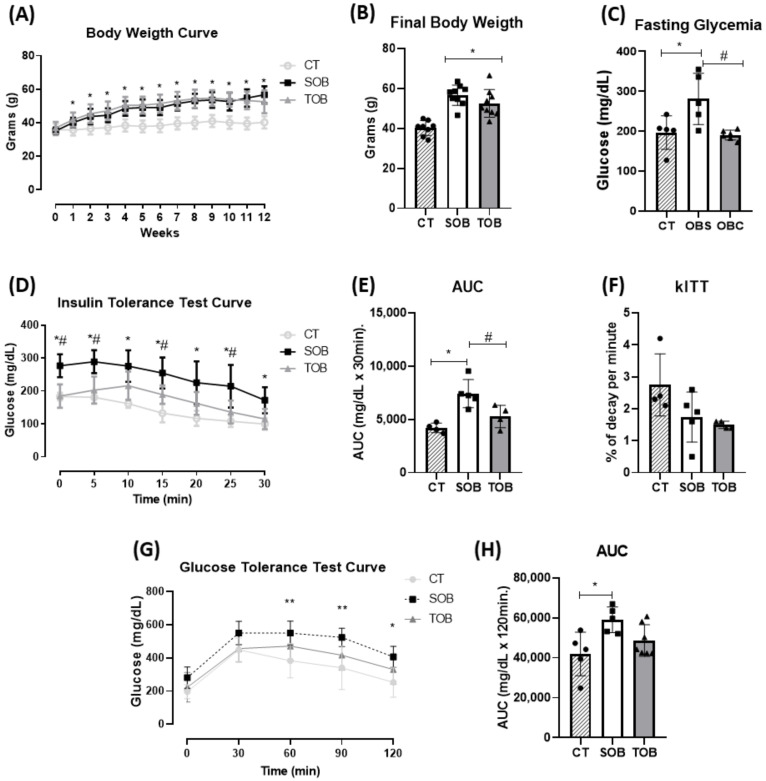
Physiological and metabolic parameters. (**A**) Body weight curve during 12 weeks of diet induction. (**B**) Final body weight. (**C**) Fasting glycemia (8 h fasting). (**D**) Glycemic curve during insulin tolerance test (ITT). (**E**) Area under the curve (AUC) during ITT. (**F**) Blood glucose decay rate (kITT) during ITT. (**G**) Glycemic curve during glucose tolerance test (GTT). (**H**) Area under the curve during GTT. The bars represent the mean ± SD of the control (CT), sedentary obese (SOB), and trained obese (TOB) groups (*n =* 26 body weight; *n* = 4–5 per group in the test). * *p* < 0.05 vs. CT; ** *p* < 0.008 vs. CT; # *p* < 0.05 vs. TOB. For the results presented in the bar graphs, comparing the three groups at a single moment, we used one-way ANOVA (**B**,**C**,**E**,**F**,**H**), followed by Bonferroni’s post-hoc test. For the line graphs, which compared the three groups at different times, we used two-way ANOVA (**A**,**D**,**G**), followed by Bonferroni’s post-hoc test.

Firstly, as expected, the high-fat diet was effective in increasing the body mass of both obese groups when compared to the control group. However, also as expected, the animals from the trained obese (TOB) group showed no difference in body weight when compared to the sedentary obese (SOB) group (Figure 1A,B—Supplementary 1A,B). The obese animals showed fasting hyperglycemia, and the exercise protocol decreased it (Figure 1C—Supplementary 1C). Moreover, during the insulin tolerance test (Figure 1D—Supplementary 1D), obese animals had a greater area under the glycemic curve and the exercise reversed this condition (Figure 1E—Supplementary 1E). No difference was found for blood glucose decay (Figure 1F—Supplementary 1F). Subsequently, the glucose tolerance test was performed. It was observed that the obese animals were more glucose-intolerant and that the short-term exercise protocol was not significantly effective in reversing this parameter (Figure 1G,H—Supplementary 1G,H).

### 2.2. Inflammatory Serum Profile and Hepatic Insulin Sensitivity

**Figure 2:**
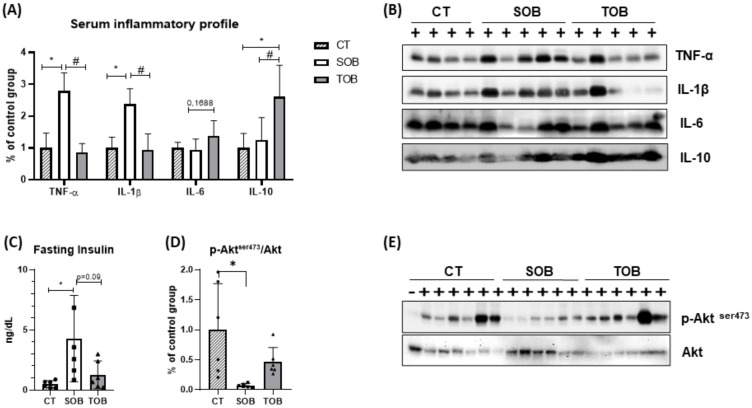
Biomolecular parameters. (**A**) Interleukin-1 beta (IL-1β), tumor necrosis factor-alpha (TNF-α), IL-6, and IL-10 content in the serum. (**B**) Band of proteins measured by immunoblotting in serum. (**C**) Fasting insulin (8 h fasting). (**D**) Phosphorylation of protein kinase B (Akt) in liver tissue. (**E**) Band of proteins measured by immunoblotting in the liver tissue. The bars represent the mean ± SD of the control (CT), sedentary obese (SOB), and trained obese groups (TOB) (*n =* 4–6 per group). * *p* < 0.05 vs. CT; # *p* < 0.05 vs. TOB. The Gaussian distribution of the serum and liver data samples was assessed using the Shapiro–Wilk test to perform Dixon’s one-sided outlier test with *p* < 0.05. One-way ANOVA non-parametric test was performed, followed by Tukey’s post-hoc test (**A**,**D**) and Bonferroni’s post-hoc test (**C**), considering *p* < 0.05 as statistical significance.

After inducing obesity and observing impaired glycemic homeostasis, we assessed the serum inflammatory profile and the liver’s insulin sensitivity of the animals. It was found that obese animals had elevated serum levels of inflammatory cytokines IL-1β and TNF-α. On the other hand, it was observed that the short period of combined physical exercise was effective in reversing these parameters, even without changing the body fat (Figure 2A,B—Supplementary 2A,B). No significant difference was found for IL-6 levels among the groups. The exercised obese group showed a slight tendency to increase. On the other hand, it was seen that combined physical exercise increased the amount of serum IL-10 in the exercised obese animals, with no difference between lean and obese (Figure 2A,B—Supplementary 2A,B). Further, the serum insulin levels and the activity of the insulin pathway in the liver were assessed. Obese animals showed hyperinsulinemia and exercised animals tended to reverse this condition (Figure 2C—Supplementary 2C). Moreover, by reducing protein kinase B (Akt) phosphorylation, we observed that obese animals had elevated liver insulin resistance. It was seen that even with higher concentrations of the hormone, obese animals presented lower Akt phosphorylation. On the other hand, the exercised obese animals showed a slight improvement in hepatic insulin sensitivity, since these animals, even with a tendency to reduce serum insulin levels, showed a tendency to increase hepatic Akt phosphorylation (Figure 2D,E—Supplementary 2D,E).

### 2.3. Inflammatory Retina Profile

**Figure 3:**
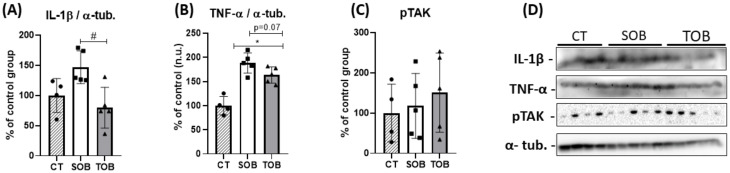
Inflammatory profile. (**A**) Quantification of IL-1β. (**B**) TNF-α content. (**C**) Phosphorylation of transforming growth factor β-activated kinase 1 (pTAK1). (**D**) Band of proteins measured by immunoblotting in retinal tissue. All proteins were relativized by α-tubulin. The bars represent the mean ± SD of the control (CT), sedentary obese (SOB), and trained obese groups (TOB) (*n =* 4–5 per group). * *p* < 0.05 vs. CT; # *p* < 0.05 vs. TOB. For the results presented in bar graphs, comparing the three groups at a single moment, we used one-way ANOVA (**A**–**C**), followed by Bonferroni’s post-hoc test. However, to assess the difference between the two obese groups (SOB and TOB) in TNF-a levels, we used Student’s *t*-test (*p* = 0.07).

We further evaluated the proinflammatory cytokines induced by obesity in response to short-term combined exercise. The animals of the sedentary obese group presented a tendentious increase of IL-1β, reversed after the combined exercise protocol, with levels below the control group (Figure 3A,D—Supplementary 3A,D). The SOB and TOB groups demonstrated a significant increase in TNF-α content and there was a tendency of exercise to reduce this parameter (Figure 3B,D—Supplementary 3B,D). Lastly, there was no significant difference in the protein content of transforming growth factor β-activated kinase 1 (pTAK1) (Figure 3C,D—Supplementary 3C,D).

## 3. Discussion

Low-grade inflammation is associated with an increase of cytokines involved in retinal tissue damage [16]. Previous studies have mainly correlated an increase of IL-1β and TNF-α levels with the emergence of several diseases [5]. Thus, finding new strategies that reduce the levels of these proinflammatory proteins is important to increase the options for the prevention and treatment of eye diseases that are triggered by inflammation. Here, we showed that short-term combined exercise was efficient in improving the inflammatory profile and, consequently, insulin sensitivity in the retina of obese mice.

Firstly, as expected, 10 weeks of obesity induction was enough to generate our scenario of interest (obesity, hyperglycemia, hyperinsulinemia, and hepatic insulin resistance). In this scenario, the combined short-term physical exercise protocol was able to reverse hyperglycemia and improve insulin secretion and its sensitivity, even without reducing body mass. These results are in accordance with the studies of Muñoz et al. and Gaspar et al. [17,18]. Further, corroborating the literature, we observed that obesity increased serum levels of proinflammatory cytokines TNF-α and IL-1β, and this improvement on inflammatory profile culminated in a lower hepatic sensitivity to insulin. On the other hand, we found that exercise was able to reduce the levels of these cytokines and that this contributed to increasing the insulin pathway activity in the liver. In the present study, obese animals did not show differences in serum IL-6 levels. This cytokine is secreted by adipose tissue and, therefore, studies suggest that IL-6 has a positive correlation with the volume of that tissue [19,20]. Contrarily, recent studies have shown that IL-6 levels are not entirely linked to the amount of body fat [21,22]. Recently, Méndez-García and collaborators, after evaluating 103 individuals, observed that overweight men and women had a reduction in the serum concentration of IL-6 and that its level also did not correlate with abdominal circumference [22]; the same was observed by Bednarek-Tupikowska and colleagues [21]. Thus, since IL-6 can play both an inflammatory and anti-inflammatory functions, depending on the conditions it is secreted [23], IL-6 levels do not seem to be the most appropriate marker for subclinical inflammation [24]. Subsequently, exercised obese animals showed a slight tendency to increase IL-6. However, the non-significant increase in this post-exercise cytokine may be to the fact that blood collection was performed 16 h after the last session. In 2010, our research group showed that serum IL-6 levels were elevated 15 min after the exercise session [9]. Finally, after evaluating the serum levels of IL-10, we observed that the exercise protocol was able to substantially increase the serum levels of IL-10 in obese animals. Therefore, as this cytokine is well known for its anti-inflammatory role [25], its elevation may have contributed to the improvement of the inflammatory profile in the serum, liver, and retina, as evidenced in the present study.

The improvement of hepatic insulin sensitivity of the exercised obese animals can be confirmed, due to the fact that these animals, even presenting a significant reduction of hyperinsulinemia, nonetheless tended to increase hepatic Akt phosphorylation and a significant reduction in hyperglycemia. This improvement in liver insulin sensitivity after exercise was also found by Pereira and colleagues [26]. In this study, the authors also used short-term exercise training to avoid altering body composition. In the end, they found that by reducing the fat accumulation and inflammation in the liver, there was an improvement of the Akt activity of obese animals.

Moreover, the proinflammatory IL-1β is a key protein that acts as the main potent mediator of ocular diseases, inducing tissue damage [27]. A recent study conducted by Tsai and colleagues evaluated the levels of vitreous cytokines in patients with diabetic retinopathy [2]. After the analyses, the authors found that IL-1β and IFN-γ (interferon-γ) levels were increased, as well as all angiogenic factors evaluated, such as fibroblast growth factor-2 (FGF2). In another study, when assessing the lacrimal cytokines profile in aniridia, Landsend and colleagues found a significant increase in the concentrations of six lacrimal cytokines, including IL-1β [28]. This increased concentration was also correlated with the meibomian gland dysfunction (MGD) parameters, including increased meibomian gland atrophy and decreased tear film functional time. In this context, when evaluating the inflammatory profile of the animals, it was possible to observe an important reduction in IL-1β protein content after the short-term combined exercise. The effects of exercise in decreasing the levels of proinflammatory cytokines are already described in the literature on other tissues such as the hypothalamus [9], muscle [7], and liver [8]. Sriwijitkamol and colleagues observed that after 8 weeks of aerobic exercise, the IκB/NF-κB pathway was inhibited, decreasing the transcription of inflammatory mediators in muscle [29]. However, thus far, no study has been performed to observe the effects of combined exercise on the retina.

In addition, IL-1β, as well as TNF-α, are responsible for the upregulation of inflammatory genes, including matrix metalloproteinase-9 (MMP-9) expression [5]. MMPs are found in almost all tissues of the eye and have been implicated in a wide range of retinal diseases and processes capable of affecting all aspects of ocular physiology [5]. In the present study, the obesity condition increased serum TNF-α and IL-1β, and the exercise protocol was sufficient to revert this parameter. Moreover, our results also showed a tendency (*p* = 0.07 SOB vs. TOB) for reduced TNF-α in the retina, which is regulated and activated by MMPs, contributing to the reduction of a causal cycle of ocular diseases [30]. In addition, it is well described in the literature that TNF-α reduction is able to attenuate insulin resistance, which in turn is the main condition for the onset of diabetes mellitus and consequently various ocular diseases [31].

On the inflammatory profile, the literature highlights the role of transforming growth factor β-activated kinase 1 (TAK-1) as a fundamental protein for propagating the inflammatory signal, since it can be activated by several ligands that interact with the transforming growth factor (TGF), B-cell receptor (BCR), TLR, AGEs (advanced glycation end-products), IL-1R, and tumor necrosis factor receptor (TNFR) receptors [32]. Moreover, TAK-1 is essential to the migration of NF-κB and AP-1 transcription factors to the nucleus [33,34]; therefore, TAK-1 reduction improves retina homeostasis. In the present study, our findings did not show any statistical difference in pTAK content between the groups. This can be explained by the levels of TNF-α and IL-1β. Significantly, in this study, only TNF-α levels were different between the control and obese groups. Thus, the TNF-α pathway alone may not be sufficient to increase the phosphorylation of TAK. Finally, as a limitation of this study, the animals’ retinal function was not assessed. In this sense, we cannot state whether or not this parameter was improved by short-term combined exercise.

In summary, due to its anti-inflammatory effect, physical exercise is an important strategy to treat and prevent obesity and its associated diseases and comorbidities. In the present study, the short period of combined training (only seven exercise sessions) seems to have contributed slightly to the reduction of inflammation in the retina, without alterations in body fatness. Here, it was possible to observe that the short training period significantly reduced levels of IL-1β and there was a tendency for the reduction of TNF-α. Therefore, these results show that a short period of combined physical exercise, even without interference from body weight, is capable of initiating a reduction in the inflammation of the retina of obese animals.

## 4. Materials and Methods

### 4.1. Animals

Four-week-old (≅20 g) Swiss mice (*Mus musculus*) were used after being approved by the Committee on Ethics in Animal Use of the State University of Campinas (UNICAMP), # 4773–1/2018 in 13/03/2018. The mice were placed in individual polyethylene cages, with an enriched environment (Polyvinyl chloride (PVC) pipes were cut in half to form a shelter 10 × 10 cm from the base and 5 cm high). The animal maintenance room was kept at 22 °C ± 2, with a relative humidity of 45–55%, on-site noises below 85 decibels, a light-dark cycle (12/12 h), and with water and a standard diet or high-fat diet (HFD) ad libitum.

### 4.2. High-Fat Diet

The mice were fed a high-fat diet (HFD) to induce obesity. The HFD was prepared according to the American Institute of Nutrition Guidelines (AIN-93G) [35] and modified to contain 35% fat (4% of soybean oil and 31% of pork lard) [36].

### 4.3. Experimental Groups

The animals were initially distributed into (i) control group (CT) (*n* = 10)—sedentary animals fed a standard diet, and obese group (OB)—sedentary animals fed an HFD for 10 weeks to induce obesity. After 10 weeks of obesity induction, the obese animals were randomly redistributed according to body weight and fasting glycemia into two groups: (ii) sedentary obese (SOB) (*n* = 11)—sedentary animals fed a HFD, and (III) trained obese (TOB) (*n* = 14)—animals fed an HFD that were subjected to the short-term combined exercise protocol. The animals were weighed weekly on an analytical balance (TE214S, Sartorius, Göttingen/Germany) during the whole experimental period.

### 4.4. Adaptation of Animals to the Exercise Apparatus

The adaptation of the animals to the exercise ladder occurred as proposed by Cassilhas and colleagues [37]. Firstly, the animals were kept inside the shelter at the top of the ladder for 60 s with an empty conical plastic tube attached to their tails. In the first attempt to climb, the animals were positioned on the ladder 15 cm from the entrance of the shelter. On the second attempt, the animals were positioned 25 cm away, while on the third attempt the animals were positioned at the base of the ladder, 70 cm from the shelter. The animals rested for 60 s when they reached the shelter. The attempts from the base of the ladder continued until the animals made three successful attempts without any stimulus [37]. The procedures were performed for five consecutive days before beginning the maximum voluntary carrying capacity (MVCC) test.

### 4.5. Determination of Maximum Voluntary Carrying Capacity (MVCC) to Prescribe the Strength Exercise Intensity

The test to determine the maximum voluntary carrying capacity (MVCC) was firstly proposed for rats and then adapted and proposed for mice [26,38]. This is a test to identify the maximum overload that each animal can carry while climbing. The animals performed the climbs carrying an apparatus (i.e., a conical plastic tube approximately 7.5 cm in height and 2.5 cm in diameter) attached with adhesive tape along their tails, where the load was placed. During the test, the animals climbed from the base of the ladder, and the attempt was considered successful when the animals reached the proposed distance of 70 cm. The first attempt was performed with an overload of 75% of the animal’s body weight, and an incremental load of 5 g was added in each new attempt to climb until the animal could no longer complete the entire course. After each successful attempt, the animals were removed from the ladder and placed in an individual cage, where they rested for 5 min until the next attempt began. The load carried in the last successful attempt was considered the MVCC, and it was used for the prescription of the individual loads in the experiment.

### 4.6. Maximum Power Determination to Prescribe the Aerobic Exercise Intensity

Firstly, the animals were adapted to the treadmill for 5 days, 10 min/day at a speed of 3 m/min. The incremental load test started with 6 m/min and 0% inclination. Exhaustion was observed when the mice touched the end of the treadmill five times in sequence in a period of less than 1 min [39]. The maximum power (Pmax.) was defined as the animal exhaust velocity (m/min) and it was used to calculate the running speed of each animal to achieve 75% of Pmax.

### 4.7. Combined Exercise Protocol

After 10 weeks of obesity induction, the animals from the TOB group started the short-term exercise protocol, which lasted 7 consecutive days. In each session, the animals initially carried out strength exercises, followed by aerobic exercises. The strength exercise protocol consisted of 10 climbing series, with 12 ± 1 dynamic climbing movements using each of the hind paws, as proposed by Frajacomo and colleagues [40]. Moreover, the animals rested for 60–90 s between the series and climbed with an overload corresponding to 70% of the MVCC. At the end of the 10 series, the animals rested for 90 s until the aerobic exercise.

**Figure 4:**
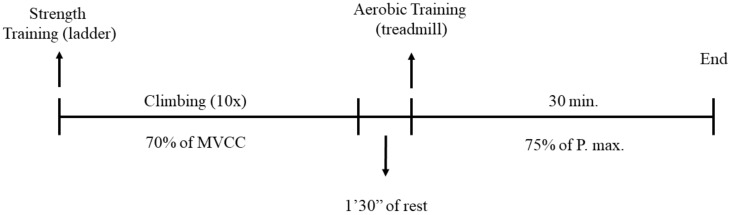
Exercise protocol. The exercise session started with a strength exercise protocol, which consisted of 10 climbs at 70% of the maximum voluntary carrying capacity (MVCC), followed by 30 min running on the treadmill at 75% of the maximum power (Pmax.).

Next, the animals were subjected to 30 min of treadmill running at an intensity of 75% of the exhaust velocity. The intensity was determined by the incremental load test, which determines the maximum power as previously described (Figure 4).

### 4.8. Insulin Tolerance Test (ITT)

The mice were subjected to the insulin tolerance test (ITT) 16 h after the fifth physical exercise session, 8 h fasting, and 12 weeks being fed the HFD. To perform the test, the mice received an intraperitoneal injection of recombinant human insulin (Humulin R) from Eli Lilly (Indianapolis, IN/USA) at a concentration of 1.5 U/kg of body weight. Blood samples were collected from the tails of the animals at 0, 5, 10, 15, 20, 25, and 30 min to determine blood glucose levels. The results were evaluated by determining the areas under the serum glucose curves (AUC) during the test by the trapezoidal method [41], using Microsoft Excel. The constant rate of plasma glucose uptake (kITT) was calculated using the formula 0.693/biological half-life (t_1/2_). Plasma glucose t_1/2_ was calculated from the slope of the least-squares analysis of serum glucose concentration during the linear decay phase [42].

### 4.9. Glucose Tolerance Test (GTT)

The mice were subjected to the glucose tolerance test (GTT) 16 h after the fifth physical exercise session, 8 h fasting, and 12 weeks being fed the HFD. After blood collection to determine the initial blood glucose, we applied a dose of 2 g/Kg glucose (intraperitoneal), and new samples were then collected from the animal’s tail after 30, 60, 90, and 120 min. The area under the curve (AUC) was calculated by glucose values mean through the trapezoidal method [41], using Microsoft Excel, Microsoft Office Professional Plus 2016, version 16.0.13029.20308

### 4.10. Collection of Biological Material

Two days after the ITT, the animals were subjected to one more exercise session, and 16 h afterwards, following 8 h of fasting, the mice were anesthetized and euthanized. For the surgical procedures, the animals were euthanized by decapitation after intraperitoneal injection of ketamine (300 mg/kg) and xylazine (30 mg/kg). Moreover, the loss of pedal and corneal reflexes were checked to ensure that the animals were anesthetized. All procedures were approved by the Committee on Ethics in Animal Use of the State University of Campinas (UNICAMP), # 4773–1/2018 in 13 March 2018.

### 4.11. Tissue Extraction and Immunoblotting Assay

After the experimental period, the animals were euthanized and the biological material was collected for molecular analysis. The blood was collected by cardiac puncture and centrifuged to separate the serum. The liver was collected and rapidly snap-frozen in liquid nitrogen and stored at −80 °C until analysis. An incision was performed with an ophthalmic scalpel, and the iris and internal ocular structures such as the vitreous and crystalline were removed. The retina was then removed with the aid of forceps and quickly frozen in liquid nitrogen for storage at −80 °C. The tissues were then prepared for Western blot analyses, according to Pereira et al. [26].

Serum samples were diluted (1:10 ratio) and 4 µL for IL-1β and TNF-α, and 20 µL for IL-6 and IL-10 were homogenized in 1× Laemmli buffer (62.5 mM Tris-HCl; pH = 6.8; 1.5% SDS; 1.5% Mercaptoethanol 2%; 0.0025% bromophenol blue (*w*/*v*); 8% glycerol (*v*/*v*)). Then, the samples were loaded into polyacrylamide gel for SDS-PAGE separation and transferred to PVDF (polyvinylidene difluoride) membranes. For the tissue, samples containing 30 μg of total protein were loaded into polyacrylamide gel for SDS-PAGE separation and transferred to PVDF (polyvinylidene difluoride) membranes. The membranes were blocked with 5% milk powder at room temperature for 1 h and incubated with primary antibodies against the protein of interest. In addition, a specific secondary antibody was used. The specific bands were labeled by chemiluminescence, and visualization was performed by the photographic documentation system in G:BOX (Syngene). The bands were quantified using the UN267 SCAN-IT gel 6.1 software. The primary antibodies used were anti-IL-6 Cell Signaling Technology (#12912); anti-IL-10 Santa Cruz Biotechnology (sc-8438); Cell Signaling Technology phospho-Akt (Ser473) (#9271), Cell Signaling Technology Akt (#4685), Cell Signaling Technology phospho-TAK1 (Ser412) (#9339), BioLegend (503502) anti-IL-1β, Cell Signaling Technology (#11948S) anti-TNF-α and anti-α-tubulin (2144) from Cell Signaling Technology (Beverly, MA, USA). The secondary antibodies used were anti-rabbit IgG antibody is conjugated to horseradish peroxidase (HRP) (7074); and anti-mouse IgG, HRP-linked 274 antibody (7076) from Cell Signaling Technology (Beverly, MA, USA).

### 4.12. Insulin Assay

Serum insulin levels were determined using a commercial kit according to the manufacturer’s instructions (Crystal Chem Ultra Sensitive Mouse Insulin ELISA Kit, #90080).

### 4.13. Statistical Analyses

All results were expressed as mean ± standard deviation of the mean (SD). The data were analyzed by Student’s *t* test when two groups were compared. One-way analysis of variance (ANOVA), followed by Bonferroni or Tukey post-hoc tests, were used to compare the control and both experimental groups. Moreover, two-way ANOVA with Bonferroni’s correction for multiple comparisons was used to compare the three groups at different moments. Statistical significance was considered when the *p* value was <0.05. The “GraphPad Prism 8.0.1.244 GraphPad Software Inc.” program was used to prepare the graphs and to perform the statistical analyses.

## Supplementary Materials

Supplementary materials can be found at https://www.mdpi.com/1422-0067/21/17/6099/s1.

## Abbreviations

AIN	American Institute of Nutrition Guidelines
AUC	Areas under the serum glucose curves
BCR	B-cell receptor
CT	Control group
FGF2	Fibroblast growth factor-2
HFD	High-fat diet
IFN-γ	Interferon-γ
IL-10	Interleukin-10
IL-1R	Interleukin-1 receptor
IL-1ra	Interleukin-1 receptor antagonist
IL-6	Interleukin-6
IL-1β	Interleukin-1 beta
ITT	Insulin tolerance test
kITT	Constant rate of plasma glucose uptake
MGD	Meibomian gland dysfunction
MMP-9	Matrix metalloproteinase-9
MVCC	Maximum voluntary carrying capacity
NF-κB	Nuclear factor-kappa B
OB	Obese group
Pmax	Maximum power
pTAK1	Transforming growth factor β-activated kinase 1
PVDF	Polyvinylidene difluoride
RAGEs	Advanced glycation end products receiver
SOB	Sedentary obese group
TGF	Transforming growth factor
TLR-4	Toll-like receptor 4
TNF-α	Tumor necrosis factor-alpha
TNFR	Tumor necrosis factor receptor
TNFsr	Soluble TNF receptor
TOB	Trained obese group
TrkB	Tropomyosin receptor kinase B
VEGF	Vascular endothelial growth factor
