# Supplementary material for: Short-Term Combined Exercise Improves Inflammatory Profile in the Retina of Obese Mice
Source: Int J Mol Sci. 2020 Aug 24;21(17):6099. doi: 10.3390/ijms21176099 (PMC7503303; doi:10.3390/ijms21176099)
Supplement: Supplementary file 1 [file ijms-21-06099-s001.pdf]

Supplementary Material

Figure 1 A. Body Weigth Curve

Body Weigth Curve

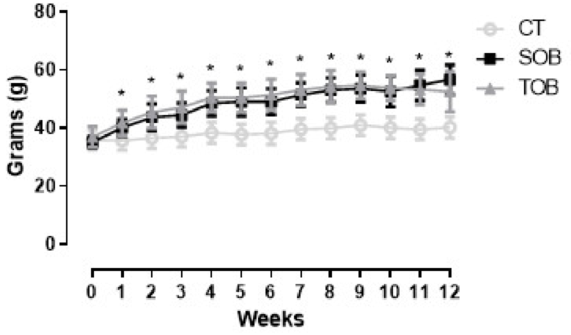

Weeks	CT							
X	A:Y1	A:Y2	A:Y3	A:Y4	A:Y5	A:Y6	A:Y7	A:Y8
0	31.2	33.1	34.3	35.2	35.9	36.8	38.1	42.5
1	30.9	32.5	35.5	33.7	36.0	37.5	38.1	41.0
2	31.6	34.1	36.3	34.1	36.7	38.8	37.1	43.5
3	31.3	35.3	36.5	34.7	36.6	38.8	40.4	42.9
4	32.3	36.6	37.3	36.4	37.8	41.0	41.6	44.0
5	32.4	37.0	38.5	34.4	37.0	41.3	38.4	43.8
6	32.3	35.9	38.0	34.4	38.4	40.8	41.9	43.7
7	33.0	37.7	41.1	36.0	41.4	41.6	41.4	44.3
8	33.5	38.3	42.0	36.5	40.7	41.9	42.3	44.5
9	34.6	39.6	41.8	37.7	40.9	43.5	44.1	45.6
10	34.3	38.4	40.8	36.5	39.2	42.6	43.8	45.1
11	33.7	39.5	40.6	35.1	39.1	41.2	43.2	44.0
12	34.2	39.5	41.0	35.8	41.3	41.0	44.2	45.0

SOB								
B:Y1	B:Y2	B:Y3	B:Y4	B:Y5	B:Y6	B:Y7	B:Y8	B:Y9
32.5	33.0	33.6	34.0	35.1	36.0	36.6	37.7	37.9
35.7	38.3	38.3	40.2	41.6	38.0	40.7	44.6	43.9
37.6	42.5	36.2	44.1	45.8	43.2	45.3	50.2	48.9
38.6	44.3	38.8	45.6	44.3	43.7	47.7	52.1	45.2
41.3	47.9	42.8	49.3	50.0	47.0	50.7	54.9	52.8
42.1	48.6	41.7	49.7	52.8	46.5	51.9	53.6	54.7
42.6	49.8	43.1	50.0	51.4	46.3	52.8	49.6	56.3
44.3	51.6	46.6	52.5	55.2	49.5	54.4	51.5	57.3
46.0	52.7	48.6	51.9	56.6	50.2	55.8	58.4	57.3
44.8	54.2	49.6	51.7	56.8	51.3	57.5	59.4	57.2
43.8	54.1	46.0	50.0	56.2	50.8	55.7	59.2	58.1
44.7	56.7	52.2	50.5	57.6	53.2	56.4	61.4	60.1
46.6	58.6	54.7	52.6	59.6	55.1	58.2	63.5	61.3

TOB								
C:Y1	C:Y2	C:Y3	C:Y4	C:Y5	C:Y6	C:Y7	C:Y8	C:Y9
30.3	34.4	35.3	35.6	36.4	38.2	40.3	41.2	40.6
35.9	38.1	37.0	41.8	41.9	41.8	47.3	44.8	47.7
40.3	42.3	40.3	41.9	41.6	46.3	52.9	48.4	55.3
42.5	44.0	41.0	43.9	45.5	46.2	54.1	49.5	57.7
45.4	46.1	45.6	50.1	50.5	49.4	55.4	51.2	60.7
46.8	45.9	46.3	49.5	50.5	48.8	54.8	50.0	62.0
46.9	47.7	48.0	50.5	50.6	49.4	56.2	50.0	63.9
48.1	48.3	52.0	53.3	51.5	50.5	57.1	51.6	65.6
48.7	49.4	53.1	55.3	52.0	52.5	58.2	53.0	66.9
50.2	50.5	52.8	54.0	55.1	52.3	59.9	53.1	64.6
49.7	46.5	53.2	54.4	55.2	50.7	57.9	58.8	57.9
48.9	49.7	50.0	54.3	54.9	49.9	57.4	49.8	65.1
43.5	49.2	47.5	53.9	55.9	50.4	58.1	48.2	66.6

Row 1					
CT vs. SOB	1.426	-1.761 to 4.613	No	ns	0.8435
CT vs. TOB	-0.3403	-3.527 to 2.847	No	ns	>0.9999
SOB vs. TOB	-1.767	-4.831 to 1.298	No	ns	0.4965
Row 2					
CT vs. SOB	-3.769	-6.956 to -0.5822	Yes	*	0.0143
CT vs. TOB	-5.436	-8.623 to -2.249	Yes	***	0.0002
SOB vs. TOB	-1.667	-4.731 to 1.398	No	ns	0.5719
Row 3					
CT vs. SOB	-6.454	-9.642 to -3.267	Yes	****	<0.0001
CT vs. TOB	-8.177	-11.36 to -4.990	Yes	****	<0.0001
SOB vs. TOB	-1.722	-4.787 to 1.342	No	ns	0.5290
Row 4					
CT vs. SOB	-6.675	-9.862 to -3.487	Yes	****	<0.0001
CT vs. TOB	-9.352	-12.54 to -6.165	Yes	****	<0.0001
SOB vs. TOB	-2.678	-5.742 to 0.3868	No	ns	0.1084
Row 5					
CT vs. SOB	-9.376	-12.56 to -6.189	Yes	****	<0.0001
CT vs. TOB	-11.34	-14.53 to -8.155	Yes	****	<0.0001
SOB vs. TOB	-1.967	-5.031 to 1.098	No	ns	0.3685
Row 6					
CT vs. SOB	-10.42	-13.61 to -7.233	Yes	****	<0.0001
CT vs. TOB	-11.86	-15.05 to -8.677	Yes	****	<0.0001
SOB vs. TOB	-1.444	-4.509 to 1.620	No	ns	0.7693
Row 7					
CT vs. SOB	-10.10	-13.29 to -6.917	Yes	****	<0.0001
CT vs. TOB	-12.47	-15.66 to -9.284	Yes	****	<0.0001
SOB vs. TOB	-2.367	-5.431 to 0.6979	No	ns	0.1911
Row 8					
CT vs. SOB	-11.06	-14.25 to -7.875	Yes	****	<0.0001
CT vs. TOB	-12.74	-15.93 to -9.553	Yes	****	<0.0001
SOB vs. TOB	-1.678	-4.742 to 1.387	No	ns	0.5631
Row 9					
CT vs. SOB	-12.30	-15.49 to -9.112	Yes	****	<0.0001
CT vs. TOB	-13.59	-16.78 to -10.40	Yes	****	<0.0001
SOB vs. TOB	-1.289	-4.353 to 1.776	No	ns	0.9332
Row 10					
CT vs. SOB	-11.87	-15.06 to -8.687	Yes	****	<0.0001
CT vs. TOB	-12.99	-16.17 to -9.798	Yes	****	<0.0001
SOB vs. TOB	-1.111	-4.176 to 1.953	No	ns	>0.9999
Row 11					
CT vs. SOB	-11.84	-15.03 to -8.657	Yes	****	<0.0001
CT vs. TOB	-13.00	-16.19 to -9.812	Yes	****	<0.0001
SOB vs. TOB	-1.156	-4.220 to 1.909	No	ns	>0.9999
Row 12					
CT vs. SOB	-14.41	-17.60 to -11.22	Yes	****	<0.0001
CT vs. TOB	-12.99	-16.17 to -9.800	Yes	****	<0.0001
SOB vs. TOB	1.422	-1.642 to 4.487	No	ns	0.7914
Row 13					
CT vs. SOB	-15.63	-18.81 to -12.44	Yes	****	<0.0001
CT vs. TOB	-11.53	-14.71 to -8.341	Yes	****	<0.0001
SOB vs. TOB	4.100	1.035 to 7.165	Yes	**	0.0043

Figure 1 B. Final Body Weigth

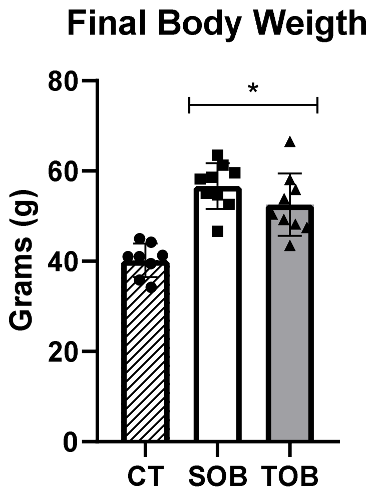

A	B	C
CT	SOB	TOB
Y	Y	Y
34.2	46.6	43.5
39.5	58.6	49.2
41.0	54.7	47.5
35.8	52.6	53.9
41.3	59.6	55.9
41.0	55.1	50.4
44.2	58.2	58.1
45.0	63.5	48.2
	61.3	66.6

P value	< 0.0001			
P value summary	***			
Are means signif. different? (P < 0.05)	Yes			
Number of groups	3			
F	20.44			
R squared	0.6399			
Bartlett's test for equal variances				
Bartlett's statistic (corrected)	2.591			
P value	0.2738			
P value summary	ns			
Do the variances differ signif. (P < 0.05)	No			
ANOVA Table	SS	df	MS	
Treatment (between columns)	1222	2	611.2	
Residual (within columns)	687.8	23	29.91	
Total	1910	25		
Bonferroni's Multiple Comparison Test	Mean Diff.	t	Significant? P < 0.05?	Summary
CT vs SOB	-16.44	6.186	Yes	***
CT vs TOB	-12.34	4.643	Yes	***
SOB vs TOB	4.100	1.590	No	ns

Figure 1 C. Fasting Glycemia

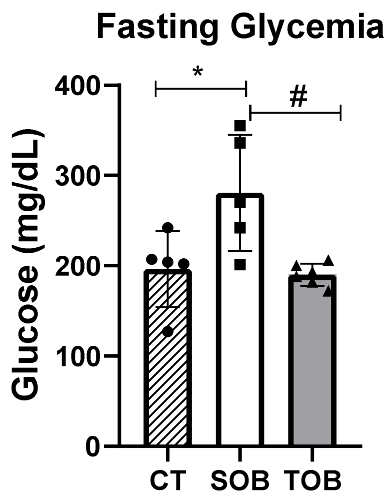

Group A	Group B	Group C
CT	SOB	TOB
202	270	193
204	355	172
242	336	182
207	201	200
127	242	206
		188

ANOVA results | Multiple comparisons

Ordinary one-way ANOVA<br>Multiple comparisons								
Number of families	1							
Number of comparisons per family	3							
Alpha	0.05							
Bonferroni's multiple comparisons test	Mean Diff.	95.00% CI of diff.	Significant?	Summary	Adjusted P Value			
CT vs. SOB	-84.40	-159.6 to -9.166	Yes	*	0.0263	A-B		
CT vs. TOB	6.233	-65.80 to 78.26	No	ns	>0.9999	A-C		
SOB vs. TOB	90.63	18.60 to 162.7	Yes	*	0.0128	B-C		
Test details	Mean 1	Mean 2	Mean Diff.	SE of diff.	n1	n2	t	DF
CT vs. SOB	196.4	280.8	-84.40	27.40	5	5	3.080	13
CT vs. TOB	196.4	190.2	6.233	26.23	5	6	0.2376	13
SOB vs. TOB	280.8	190.2	90.63	26.23	5	6	3.455	13

Figure 1 D. Insulin Tolerance Teste Curve

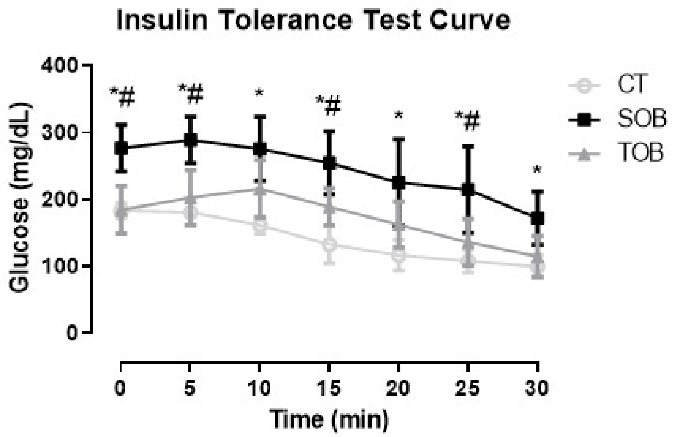

Time	CT					SOB				
X	A:Y1	A:Y2	A:Y3	A:Y4	A:Y5	B:Y1	B:Y2	B:Y3	B:Y4	B:Y5
0	200	173	180	181		293	245	284	239	323
5	185	163	170	206		286	261	268	282	349
10	152	149	171	175		293	222	250	266	348
15	93	133	149	156		282	201	229	241	321
20	86	115	126	140		196	165	210	221	335
25	84	114	110	124		168	152	292	185	277
30	76	101	104	118		160	132	182	151	235

TOB			
C:Y1	C:Y2	C:Y3	C:Y4
180	233	147	178
224	244	149	194
245	259	166	195
199	222	157	178
184	196	120	150
161	163	88	133
126	135	69	130

Bonferroni's multiple comparisons test	Predicted (LS) mean diff.	95.00% CI of diff.	Significant?	Summary	Adjusted P Value
Row 1					
CT vs. SOB	-93.30	-157.3 to -29.31	Yes	**	0.0023
CT vs. TOB	-1.000	-68.45 to 66.45	No	ns	>0.9999
SOB vs. TOB	92.30	28.31 to 156.3	Yes	**	0.0025
Row 2					
CT vs. SOB	-108.2	-172.2 to -44.21	Yes	***	0.0004
CT vs. TOB	-21.75	-89.20 to 45.70	No	ns	>0.9999
SOB vs. TOB	86.45	22.46 to 150.4	Yes	**	0.0049
Row 3					
CT vs. SOB	-114.0	-178.0 to -50.06	Yes	***	0.0002
CT vs. TOB	-54.50	-122.0 to 12.95	No	ns	0.1511
SOB vs. TOB	59.55	-4.440 to 123.5	No	ns	0.0757

Row 4					
CT vs. SOB	-122.0	-186.0 to -58.06	Yes	****	<0.0001
CT vs. TOB	-56.25	-123.7 to 11.20	No	ns	0.1311
SOB vs. TOB	65.80	1.810 to 129.8	Yes	*	0.0420
Row 5					
CT vs. SOB	-108.6	-172.6 to -44.66	Yes	***	0.0004
CT vs. TOB	-45.75	-113.2 to 21.70	No	ns	0.2945
SOB vs. TOB	62.90	-1.090 to 126.9	No	ns	0.0554
Row 6					
CT vs. SOB	-106.8	-170.8 to -42.81	Yes	***	0.0005
CT vs. TOB	-28.25	-95.70 to 39.20	No	ns	0.9068
SOB vs. TOB	78.55	14.56 to 142.5	Yes	*	0.0115
Row 7					
CT vs. SOB	-72.25	-136.2 to -8.260	Yes	*	0.0222
CT vs. TOB	-15.25	-82.70 to 52.20	No	ns	>0.9999
SOB vs. TOB	57.00	-6.990 to 121.0	No	ns	0.0953

Figure 1 E. AUC-ITT

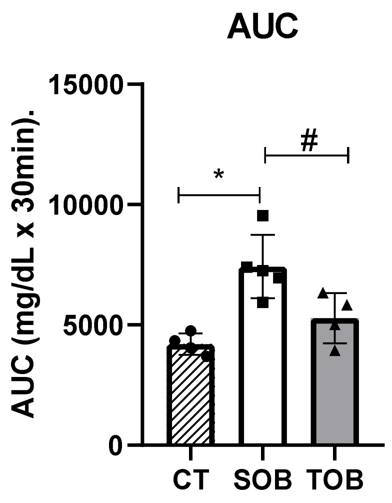

CT	SOB	TOB
Y	Y	Y
3690.0	7257.5	5830
4055.0	5947.5	6340
4340.0	7410.0	3940
4752.5	6950.0	5020
	9545.0	

Table Analyzed	AUC				
One-way analysis of variance					
P value	0.0028				
P value summary	**				
Are means signif. different? (P < 0.05	Yes				
Number of groups	3				
F	11.24				
R squared	0.6921				
ANOVA Table	SS	df	MS		
Treatment (between columns)	24340000	2	12170000		
Residual (within columns)	10830000	10	1083000		
Total	35160000	12			
Bonferroni's Multiple Comparison Test	Mean Diff.	t	Significant? P < 0.05?	Summary	95% CI of diff
CT vs SOB	-3213	4.603	Yes	**	-5216 to -1209
CT vs TOB	-1073	1.459	No	ns	-3185 to 1039
SOB vs TOB	2140	3.065	Yes	*	136.2 to 4143

Figure 1 F. kITT

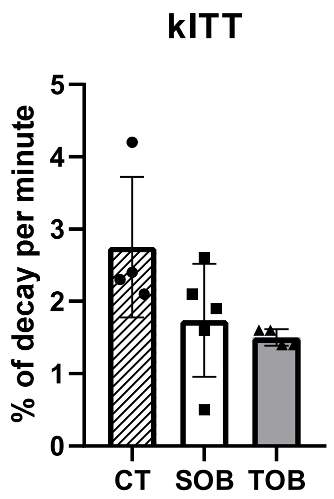

CT	SOB	TOB
4.200000	2.100000	1.400000
2.400000	2.600000	1.600000
2.100000	1.900000	1.400000
2.300000	1.600000	1.600000
	0.500000	

Table Analyzed	kITT - 0,693 / t/2kITT				
One-way analysis of variance					
P value	0.0769				
P value summary	ns				
Are means signif. different? (P < 0.05)	No				
Number of groups	3				
F	3.352				
R squared	0.4013				
ANOVA Table	SS	df	MS		
Treatment (between columns)	3.581	2	1.791		
Residual (within columns)	5.342	10	0.5342		
Total	8.923	12			
Bonferroni's Multiple Comparison Test	Mean Diff.	t	Significant? P < 0.05?	Summary	95% CI of diff
CT vs SOB	1.010	2.060	No	ns	-0.3972 to 2.417
CT vs TOB	1.250	2.419	No	ns	-0.2333 to 2.733
SOB vs TOB	0.2400	0.4895	No	ns	-1.167 to 1.647

Figure 1 G. Glucose Tolerance Teste Curve

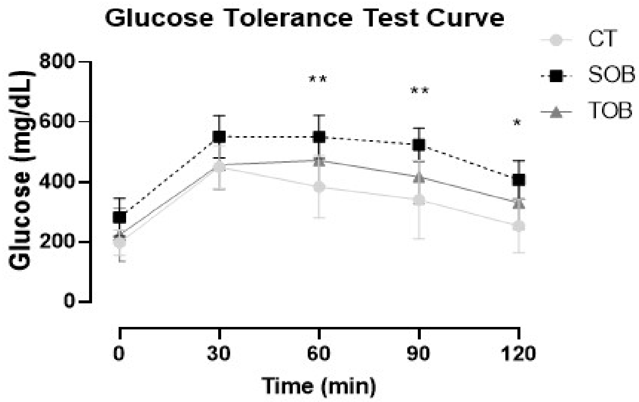

Time	CT							SOB				
X	A:Y1	A:Y2	A:Y3	A:Y4	A:Y5	A:Y6	A:Y7	B:Y1	B:Y2	B:Y3	B:Y4	B:Y5
0	202	204	242	207	127			270	355	336	201	242
30	468	493	519	431	336			600	600	504	449	600
60	354	455	486	402	221			600	600	434	526	591
90	296	375	497	388	144			565	600	477	482	497
120	190	304	342	301	128			424	509	385	355	357

TOB						
C:Y1	C:Y2	C:Y3	C:Y4	C:Y5	C:Y6	C:Y7
193	172	424	182	200	206	188
479	461	600	386	471	342	457
596	406	517	415	553	371	440
600	321	425	367	420	427	355
504	266	366	303	262	350	259

Tabular results ×   Multiple

Mixed-effects analysis
Multiple comparisons

7	Bonferroni's multiple comparisons test	Predicted (LS) mean diff.	95.00% CI of diff.	Significant?	Summary	Adjusted P Value
8						
9	Row 1					
10	SOB vs. CT	84.40	-46.84 to 215.6	No	ns	0.3477
11	TOB vs. CT	27.17	-94.33 to 148.7	No	ns	>0.9999
12	TOB vs. SOB	-57.23	-178.7 to 64.27	No	ns	0.7385
13						
14	Row 2					
15	SOB vs. CT	101.2	-30.04 to 232.4	No	ns	0.1833
16	TOB vs. CT	7.171	-114.3 to 128.7	No	ns	>0.9999
17	TOB vs. SOB	-94.03	-215.5 to 27.47	No	ns	0.1807
18						
19	Row 3					
20	SOB vs. CT	166.6	35.36 to 297.8	Yes	**	0.0087
21	TOB vs. CT	87.54	-33.96 to 209.0	No	ns	0.2380
22	TOB vs. SOB	-79.06	-200.6 to 42.44	No	ns	0.3355
23						
24	Row 4					
25	SOB vs. CT	184.2	52.96 to 315.4	Yes	**	0.0034
26	TOB vs. CT	76.43	-45.07 to 197.9	No	ns	0.3716
27	TOB vs. SOB	-107.8	-229.3 to 13.73	No	ns	0.0972
28						
29	Row 5					
30	SOB vs. CT	153.0	21.76 to 284.2	Yes	*	0.0175
31	TOB vs. CT	77.00	-44.50 to 198.5	No	ns	0.3635
32	TOB vs. SOB	-76.00	-197.5 to 45.50	No	ns	0.3777

Figure 1 H. AUC – GTT

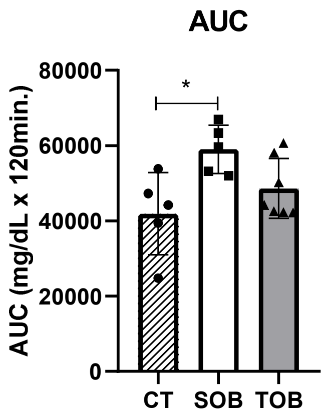

Group A	Group B	Group C
CT	SOB	TOB
39420	63360	60705
47310	66960	42210
53820	53265	58110
44250	52050	42315
24855	59625	50250
		42540
		44265

ANVOA results | Multiple comparisons

Ordinary one-way ANOVA<br>Multiple comparisons								
Number of families	1							
Number of comparisons per family	3							
Alpha	0.05							
**Bonferroni's multiple comparisons test**	**Mean Diff.**	**95.00% CI of diff.**	**Significant?**	**Summary**	**Adjusted P Value**			
CT vs. SOB	-17121	-31742 to -2500	Yes	*	0.0199	A-B		
CT vs. TOB	-6697	-20234 to 6840	No	ns	0.6005	A-C		
SOB vs. TOB	10424	-3113 to 23961	No	ns	0.1652	B-C		
**Test details**	**Mean 1**	**Mean 2**	**Mean Diff.**	**SE of diff.**	**n1**	**n2**	**t**	**DF**
CT vs. SOB	41931	59052	-17121	5380	5	5	3.182	14
CT vs. TOB	41931	48628	-6697	4981	5	7	1.345	14
SOB vs. TOB	59052	48628	10424	4981	5	7	2.093	14

Figure 2 A.

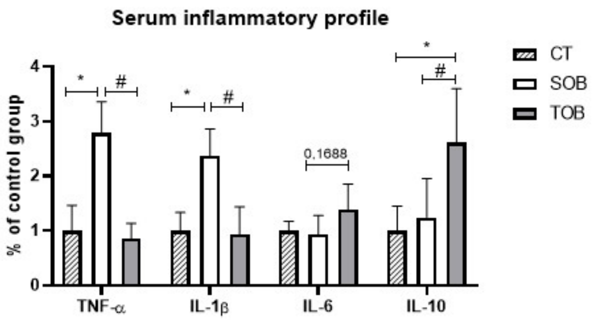

Fig. 2B

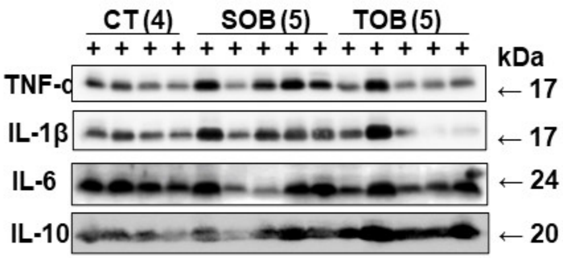

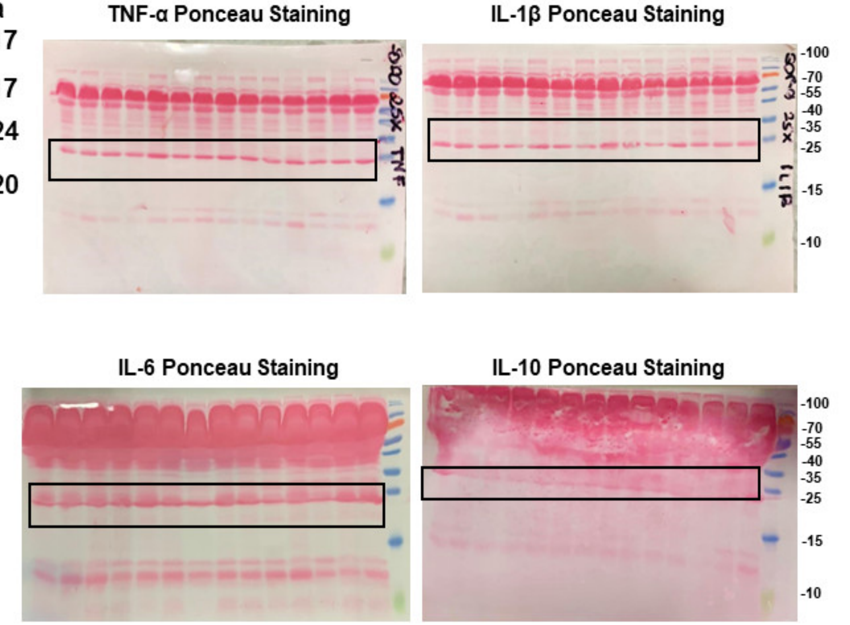

Densitometry	
**TNF**	**IL1B**
6.758.820	5.822.770
7.116.113	6.972.941
3.562.406	3.952.234
2.448.820	3.409.820
16.344.134	14.502.719
1.380.335	3.721.820
11.677.598	10.900.719
16.235.426	10.356.841
11.192.355	9.212.134
4.888.234	7.088.477
17.554.841	19.935.376
2.750.820	2.390.991
3.660.234	478.749
5.829.184	1.646.062

Normalization	
**TNF**	**IL1B**
1,359502355	1,155439604
1,43137003	1,383673438
0,716559895	0,784260358
0,49256772	0,6766266
3,28753964	2,877842658
2,348889597	2,163080877
3,265673577	2,055156611
2,251285429	1,828007024
0,983243471	1,406599789
0,553313488	0,474455576
0,736237501	0,095000413
1,17251079	0,326635815

Densidometry	Normalization
IL-6	9152,428
9874,024	1,07884203
11057,782	1,208180168
7728,296	0,844398448
7949,61	0,868579354
11795,903	1,28882773
6300,953	0,68844606
4198,054	0,458682002
9569,075	1,045523111
10737,539	1,173190218
9238,439	1,009397616
15539,681	1,697875252
8782,317	0,959561441
10989,995	1,200773718
18734,167	2,046906788

Densidometry	Normalization
IL-10	4489,671
5456,598	1,215367006
6458,376	1,438496496
4287,355	0,954937455
1756,355	0,391199043
5911,184	1,316618523
781,577	0,174083357
5132,113	1,143093336
9522,113	2,120893268
6623,355	1,475242841
8618,012	1,919519715
18617,326	4,146701618
9276,062	2,066089475
8302,184	1,849174249
13735,912	3,05944734

Ordinary one-way ANOVA
ANOVA results

Table Analyzed	TNF SORO (4x5x				
Data sets analyzed	A-C				
**ANOVA summary**					
F	22.67				
P value	0.0003				
P value summary	***				
Significant diff. among means (P < 0.05)?	Yes				
R square	0.8344				
**Brown-Forsythe test**					
F (DFn, DFd)	9.212 (2, 9)				
P value	0.0066				
P value summary	**				
Are SDs significantly different (P < 0.05)?	Yes				
**Bartlett's test**					
Bartlett's statistic (corrected)	1.296				
P value	0.5231				
P value summary	ns				
Are SDs significantly different (P < 0.05)?	No				
**ANOVA table**	**SS**	**DF**	**MS**	**F (DFn, DFd)**	**P value**
Treatment (between columns)	9.241	2	4.621	F (2, 9) = 22.67	P=0.0003
Residual (within columns)	1.834	9	0.2038		
Total	11.08	11			
**Data summary**					
Number of treatments (columns)	3				
Number of values (total)	12				

Ordinary one-way ANOVA
ANOVA results

Table Analyzed	IL-6				
Data sets analyzed	A-C				
**ANOVA summary**					
F	2.182				
P value	0.1592				
P value summary	ns				
Significant diff. among means (P < 0.05)?	No				
R square	0.2840				
**Brown-Forsythe test**					
F (DFn, DFd)	0.8554 (2, 11)				
P value	0.4516				
P value summary	ns				
Are SDs significantly different (P < 0.05)?	No				
**Bartlett's test**					
Bartlett's statistic (corrected)	2.470				
P value	0.2908				
P value summary	ns				
Are SDs significantly different (P < 0.05)?	No				
**ANOVA table**	**SS**	**DF**	**MS**	**F (DFn, DFd)**	**P value**
Treatment (between columns)	0.5810	2	0.2905	F (2, 11) = 2.182	P=0.1592
Residual (within columns)	1.465	11	0.1332		
Total	2.046	13			
**Data summary**					
Number of treatments (columns)	3				
Number of values (total)	14				

Ordinary one-way ANOVA
ANOVA results

Table Analyzed	IL1B SORO (4x5x5)				
Data sets analyzed	A-C				
**ANOVA summary**					
F	13.07				
P value	0.0022				
P value summary	**				
Significant diff. among means (P < 0.05)?	Yes				
R square	0.7438				
**Brown-Forsythe test**					
F (DFn, DFd)	0.3147 (2, 9)				
P value	0.7377				
P value summary	ns				
Are SDs significantly different (P < 0.05)?	No				
**Bartlett's test**					
Bartlett's statistic (corrected)	0.5042				
P value	0.7772				
P value summary	ns				
Are SDs significantly different (P < 0.05)?	No				
**ANOVA table**	**SS**	**DF**	**MS**	**F (DFn, DFd)**	**P value**
Treatment (between columns)	5.274	2	2.637	F (2, 9) = 13.07	P=0.0022
Residual (within columns)	1.816	9	0.2018		
Total	7.090	11			
**Data summary**					
Number of treatments (columns)	3				
Number of values (total)	12				

Ordinary one-way ANOVA
ANOVA results

Table Analyzed	il-10				
Data sets analyzed	A-C				
**ANOVA summary**					
F	5.995				
P value	0.0173				
P value summary	*				
Significant diff. among means (P < 0.05)?	Yes				
R square	0.5215				
**Brown-Forsythe test**					
F (DFn, DFd)	0.3996 (2, 11)				
P value	0.6800				
P value summary	ns				
Are SDs significantly different (P < 0.05)?	No				
**Bartlett's test**					
Bartlett's statistic (corrected)	1.685				
P value	0.4306				
P value summary	ns				
Are SDs significantly different (P < 0.05)?	No				
**ANOVA table**	**SS**	**DF**	**MS**	**F (DFn, DFd)**	**P value**
Treatment (between columns)	7.095	2	3.547	F (2, 11) = 5.995	P=0.0173
Residual (within columns)	6.509	11	0.5917		
Total	13.60	13			
**Data summary**					
Number of treatments (columns)	3				
Number of values (total)	14				

Figure 2 C. Fasting Insulin

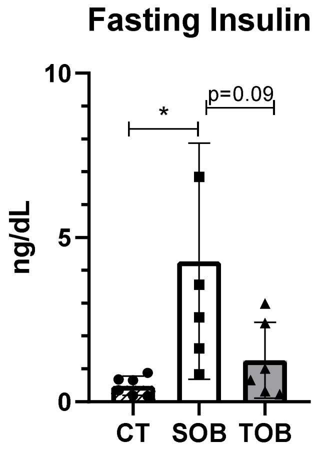

Group A	Group B	Group C
CT	SOB	TOB
0.650391	10.240230	0.318359
0.347656	1.617188	0.230469
0.191406	0.835938	2.984375
0.171875	2.574219	2.398438

ANOVA results ×

Multiple comparisons

**Ordinary one-way ANOVA** Multiple comparisons								
Number of families	1							
Number of comparisons per family	3							
Alpha	0.05							
**Bonferroni's multiple comparisons test**	**Mean Diff.**	**95.00% CI of diff.**	**Significant?**	**Summary**	**Adjusted P Value**			
CT vs. OBS	-3.792	-7.189 to -0.3961	Yes	*	0.0265	A-B		
CT vs. OBC	-0.7796	-4.176 to 2.617	No	ns	>0.9999	A-C		
OBS vs. OBC	3.013	-0.3835 to 6.409	No	ns	0.0913	B-C		
**Test details**	**Mean 1**	**Mean 2**	**Mean Diff.**	**SE of diff.**	**n1**	**n2**	**t**	**DF**
CT vs. OBS	0.4860	4.278	-3.792	1.261	6	6	3.008	15
CT vs. OBC	0.4860	1.266	-0.7796	1.261	6	6	0.6184	15
OBS vs. OBC	4.278	1.266	3.013	1.261	6	6	2.390	15

Figure 2 D. pAkt^{ser473}/Akt

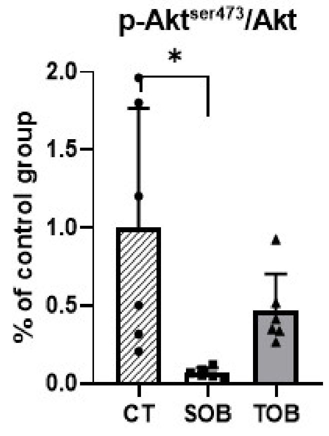

Fig.2E

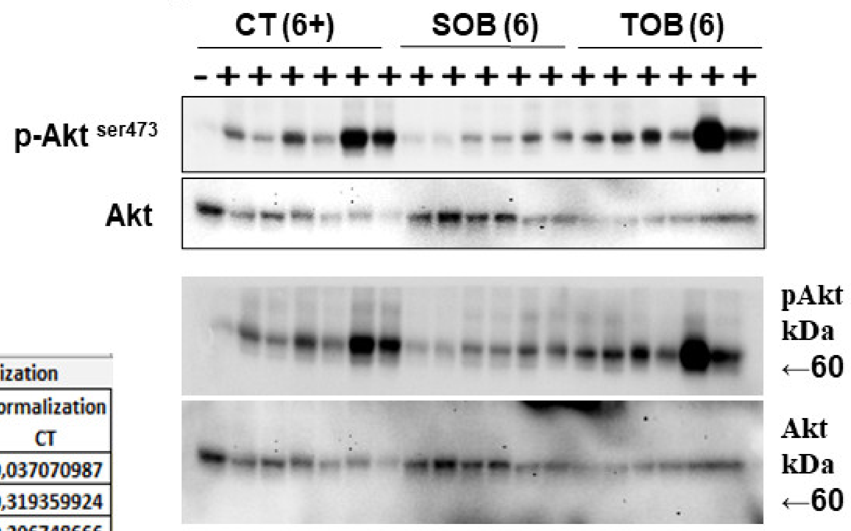

pAkt Ponceau Staining

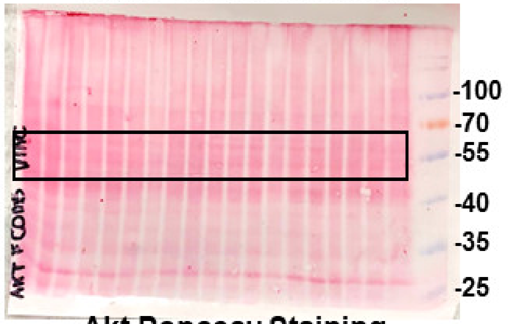

Akt Ponceau Staining

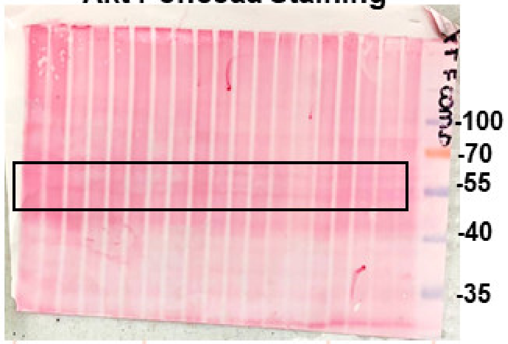

Densitometry		
p-Akt	Akt	vinc
641.314	3.274.820	3.349.527
2.169.335	1.285.870	4.701.284
1.524.213	1.395.577	5.343.991
3.134.456	1.175.920	2.032.991
1.999.042	314.263	2.024.698
6.006.092	630.749	1.269.163
4.525.577	436.506	1.429.284
840.556	3.285.991	1.583.456
780.021	4.724.163	1.633.698
1.125.314	3.966.577	1.701.456
1.196.021	3.290.284	2.863.113
1.680.556	2.598.042	1.890.991
1.564.092	3.256.627	1.045.749
2.555.698	1.823.506	5.237.820
3.146.163	1.723.335	1.331.920
3.134.042	1.151.263	1.111.456
2.185.385	1.234.799	1.648.698
10.271.113	2.108.284	543.870
4.740.991	2.184.385	3.583.941

Normalization	
p-Akt/Akt	normalization CT
0,195832	0,037070987
1,687056	0,319359924
1,092174	0,206748666
2,665535	0,504586071
6,361048	1,204147044
9,522159	1,802545627
10,36773	1,962612669
0,2558	0,048422938
0,165113	0,031255919
0,283699	0,053704254
0,363501	0,068810748
0,646855	0,122449687
0,48028	0,090917007
1,40153	0,26530974
1,825625	0,345590955
2,722264	0,515324893
1,769831	0,335029112
4,871788	0,922230012
2,170401	0,410857106

Number of families	1							
Number of comparisons per family	3							
Alpha	0.05							
**Tukey's multiple comparisons test**	**Mean Diff.**	**95.00% CI of diff.**	**Significant?**	**Summary**	**Adjusted P Value**			
CT vs. SOB	0.9307	0.2336 to 1.628	Yes	**	0.0091	A-B		
CT vs. TOB	0.5343	-0.1628 to 1.231	No	ns	0.1487	A-C		
SOB vs. TOB	-0.3965	-1.094 to 0.3007	No	ns	0.3291	B-C		
**Test details**	**Mean 1**	**Mean 2**	**Mean Diff.**	**SE of diff.**	**n1**	**n2**	**q**	**DF**
CT vs. SOB	1.000	0.06926	0.9307	0.2684	6	6	4.904	15
CT vs. TOB	1.000	0.4657	0.5343	0.2684	6	6	2.815	15
SOB vs. TOB	0.06926	0.4657	-0.3965	0.2684	6	6	2.089	15

Figure 3 A. IL-1β

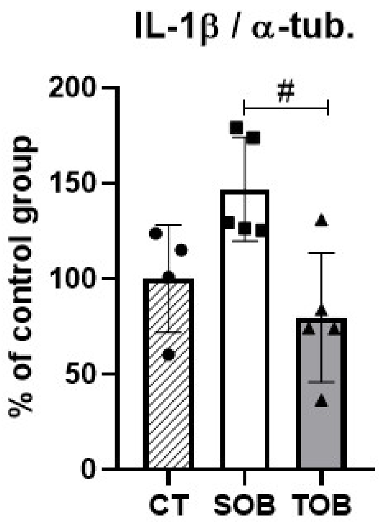

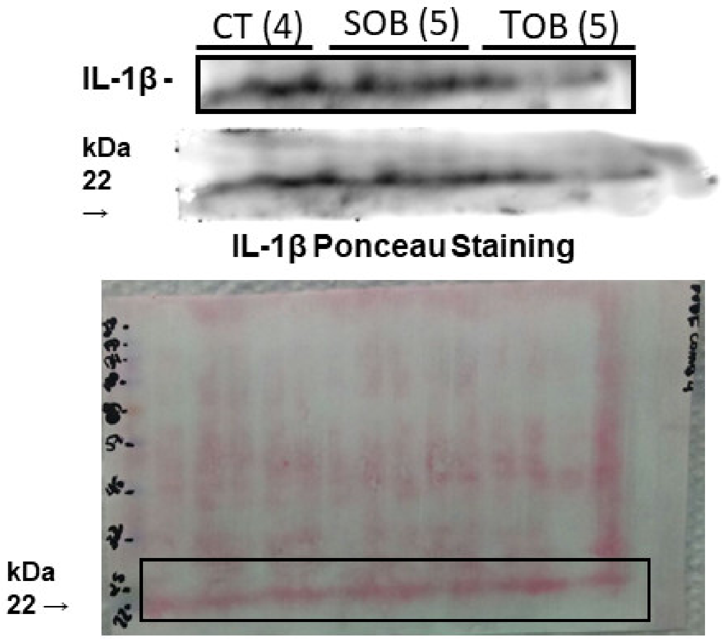

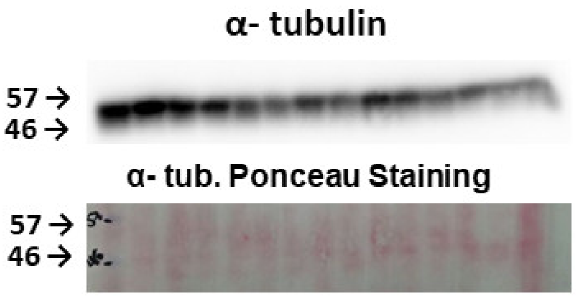

Quantification/Normalization

CT				SOB				TOB			
IL-1B	a-tub	IL-1B/a-tub	Normalized	IL-1B	a-tub	IL-1B/a-tub	Normalized	IL-1B	a-tub	IL-1B/a-tub	Normalized
35232	293630	0,11998774	60,24409987	58194	225671	0,25787097	129,4732662	59491	228269	0,26061796	130,8524868
64507	321098	0,20089505	100,8664862	68358	197301	0,34646555	173,9553175	28869	196313	0,14705598	73,83466832
70582	286240	0,24658329	123,8058838	57146	228913	0,24964069	125,3409631	13266	183180	0,07242057	36,36131541
59191	258238	0,22921104	115,0835302	71778	201113	0,35690383	179,1962249	24454	146189	0,16727661	83,98715307
Mean		0,19916928		62295	247376	0,25182314	126,4367357	20480	138836	0,14751217	74,06371755

Prism Analysis

Table Analyzed	IL1B
Column B	SOB
vs	vs
Column C	TOB
Unpaired t test	
P value	0.0087
P value summary	**
Are means signif. different? (P < 0.05)	Yes
One- or two-tailed P value?	Two-tailed
t, df	t=3.453 df=8
How big is the difference?	
Mean ± SEM of column B	146.9 ± 12.17 N=5
Mean ± SEM of column C	79.82 ± 15.13 N=5
Difference between means	67.06 ± 19.42
95% confidence interval	22.28 to 111.8
R squared	0.5985
F test to compare variances	
F,DFn, Dfd	1.546, 4, 4
P value	0.6833
P value summary	ns
Are variances significantly different?	No

Figure 3 B. TNF-α

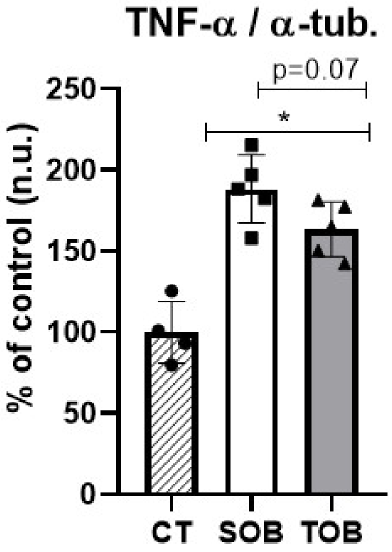

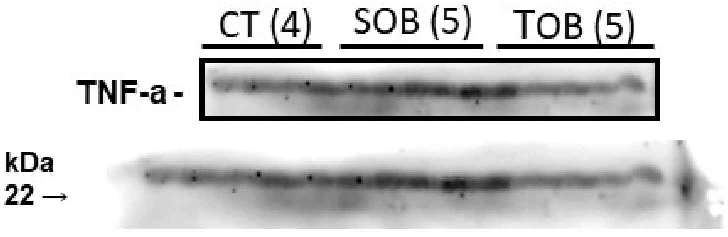

TNF-α- Ponceau Staining

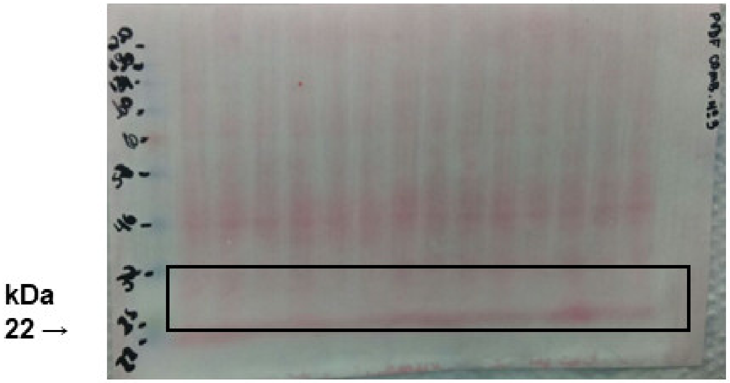

α- tubulin

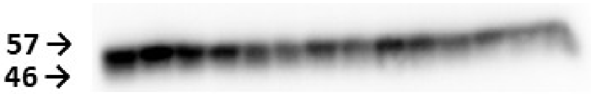

α- tub. Ponceau Staining

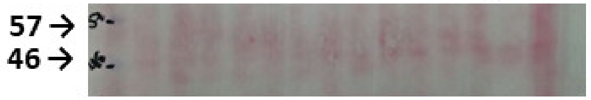

Prism Analysis

Table Analyzed	TNF-a
Column B	SOB
vs	vs
Column C	TOB
Unpaired t test	
P value	0.0726
P value summary	ns
Are means signif. different? (P < 0.05)	No
One- or two-tailed P value?	Two-tailed
t, df	t=2.067 df=8
How big is the difference?	
Mean ± SEM of column B	188.4 ± 9.363 N=5
Mean ± SEM of column C	163.6 ± 7.548 N=5
Difference between means	24.86 ± 12.03
95% confidence interval	-2.874 to 52.59
R squared	0.3481
F test to compare variances	
F,DFn, Dfd	1.539, 4, 4
P value	0.6865
P value summary	ns
Are variances significantly different?	No

Quantification/Normalization

CTS				SOB				TOB			
TNF-a	a-tub	TNF-a/a-tub	Normalized	TNF-a	a-tub	TNF-a/a-tub	Normalized	TNF-a	a-tub	TNF-a/a-tub	Normalized
61641	293630	0,20992746	79,96573149	93799	225671	0,4156449	158,3277787	108900	228269	0,47706872	181,725389
78747	321098	0,24524289	93,41811093	111677	197301	0,56602349	215,6101076	73506	196313	0,37443267	142,6291829
75939	286240	0,26529835	101,0576545	113190	228913	0,49446733	188,3528807	72430	183180	0,39540343	150,6173819
85120	258238	0,32961841	125,558503	104095	201113	0,51759459	197,1625329	63410	146189	0,43375357	165,2257461
Mean		0,26252178		118695	247376	0,47981615	182,7719417	64781	138836	0,46660088	177,7379713

Figure 3 C. TAK

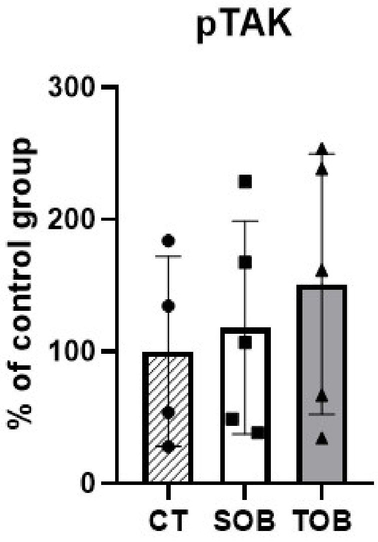

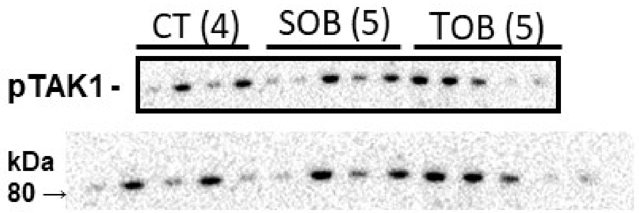

pTAK-Ponceau Staining

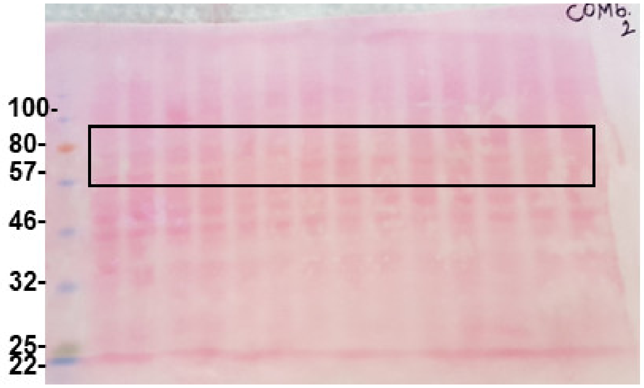

α- tubulin

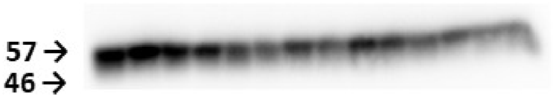

α- tub. Ponceau Staining

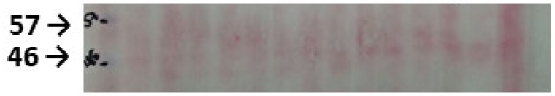

Prism Analysis

Table Analyzed	pTak				
Data sets analyzed	A-C				
**ANOVA summary**					
F	0.4180				
P value	0.6684				
P value summary	ns				
Significant diff. among means (P < 0.05)?	No				
R square	0.07063				
**Brown-Forsythe test**					
F (DFn, DFd)	0.3041 (2, 11)				
P value	0.7438				
P value summary	ns				
Are SDs significantly different (P < 0.05)?	No				
**Bartlett's test**					
Bartlett's statistic (corrected)	0.3268				
P value	0.8493				
P value summary	ns				
Are SDs significantly different (P < 0.05)?	No				
**ANOVA table**	**SS**	**DF**	**MS**	**F (DFn, DFd)**	**P value**
Treatment (between columns)	6114	2	3057	F (2, 11) = 0.4180	P=0.6684
Residual (within columns)	80446	11	7313		
Total	86560	13			
**Data summary**					
Number of treatments (columns)	3				
Number of values (total)	14				

Quantification/Normalization

CT				SOB			
pTAK	a-tub	pTAK/a-tub	Normalized	pTAK	a-tub	pTAK/a-tub	Normalized
10287	293630	0,03503389	27,84465708	13862	225671	0,0614257	48,82066606
54259	321098	0,16897956	134,3036278	9517	197301	0,04823594	38,33754881
19432	286240	0,06788709	53,9561232	65883	228913	0,28780803	228,7475592
59750	258238	0,23137571	183,8955919	27062	201113	0,13456117	106,9481569
Mean		0,12581906		52120	247376	0,21069142	167,4558805

TOB			
pTAK	a-tub	pTAK/a-tub	Normalized
68447	228269	0,29985237	238,3203021
62661	196313	0,31918925	253,6891075
37236	183180	0,20327547	161,5617415
6342	146189	0,0433822	34,47982885
11678	138836	0,08411363	66,85285167
